# Anti-CD20 antibodies and bendamustine attenuate humoral immunity to COVID-19 vaccination in patients with B-cell non-Hodgkin lymphoma

**DOI:** 10.1007/s00277-023-05204-7

**Published:** 2023-04-12

**Authors:** Takashi Ishio, Shihori Tsukamoto, Emi Yokoyama, Koh Izumiyama, Makoto Saito, Haruna Muraki, Mirei Kobayashi, Akio Mori, Masanobu Morioka, Takeshi Kondo

**Affiliations:** 1 Blood Disorders Center, Aiiku Hospital, Sapporo, Japan; 2 Division of Laboratory, Aiiku Hospital, Sapporo, Japan

**Keywords:** COVID-19 vaccine, SARS-CoV-2, B-cell non-Hodgkin lymphoma (B-NHL), Anti-CD20 antibodies, Bendamustine

## Abstract

Serologic responses of COVID-19 vaccine are impaired in patients with B-cell lymphoma, especially those who had recently been treated with anti-CD20 monoclonal antibodies. However, it is still unclear whether those patients develop an immune response following vaccination. We investigated the efficacy of vaccination against SARS-CoV-2 in 171 patients with B-cell non-Hodgkin lymphoma (B-NHL) who received two doses of an mRNA-based COVID-19 vaccine and we compared the efficacy of vaccination to that in 166 healthy controls. Antibody titers were measured 3 months after administration of the second vaccine dose. Patients with B-NHL showed a significantly lower seroconversion rate and a lower median antibody titer than those in healthy controls. The antibody titers showed correlations with the period from the last anti-CD20 antibody treatment to vaccination, the period from the last bendamustine treatment to vaccination and serum IgM level. The serologic response rates and median antibody titers were significantly different between diffuse large B-cell lymphoma (DLBCL) patients in whom anti-CD20 antibody treatment was completed within 9 months before vaccination and follicular lymphoma (FL) patients in whom anti-CD20 antibody treatment was completed within 15 months before vaccination. Moreover, the serologic response rates and median antibody titers were significantly different among FL patients in whom bendamustine treatment was completed within 33 months before vaccination. We demonstrated that B-NHL patients who were recently treated with anti-CD20 antibodies and bendamustine had a diminished humoral response to COVID-19 vaccination. UMIN 000,045,267.

**Supplementary Information:**

The online version contains supplementary material available at 10.1007/s00277-023-05204-7.

## Introduction

The novel coronavirus disease 2019 (COVID-19), caused by severe acute respiratory syndrome coronavirus 2 (SARS-CoV-2), has led to a pandemic throughout the world and more than 5 million deaths. COVID-19 has an increased risk of mortality in patients with hematological malignancies, with a mortality rate of approximately 35% in hospitalized patients, probably due to impaired humoral and cellular immunity and therapy-related immunosuppression [1–11]. The results of several randomized trials for BNT162b2 (Pfizer-BioNTech) and mRNA-1273 (Moderna) vaccines against SARS-CoV-2 have shown that the vaccines are safe and effective for preventing infection or attenuating disease severity [12, 13]. It was reported that seroconversion of SARS-CoV-2 immunoglobulin G (IgG) occurred in almost all healthy individuals, but the seroconversion rate was lower in patients with hematological malignancies [14–22]. Moreover, several studies have shown that the seroconversion rate was decreased in patients with B-cell lymphoma, especially those who had recently been treated with anti-CD20 monoclonal antibodies due to depletion of normal B cells and thus impairment of humoral response [23–30]. However, it is still unclear whether those patients develop an immune response following vaccination. In the current study, we investigated the antibody titers of SARS-CoV-2 in patients with B-cell non-Hodgkin lymphoma (B-NHL) who received two doses of an mRNA-based COVID-19 vaccine, either BNT162b2 or mRNA-1273, and compared them to those in healthy controls to identify factors affecting the response rate to the vaccine.

## Methods

### Patients and healthy controls

Previously treated, actively treated at the time of vaccination, and treatment-naïve B-NHL patients, including patients with diffuse large B-cell lymphoma (DLBCL) and patients with follicular lymphoma (FL), were enrolled in this prospective study (UMIN 000,045,267). Consistent with our previous report, all patients who had been vaccinated with two consecutive doses of an mRNA-based COVID-19 vaccine, either BNT162b2 or mRNA-1273, were recruited into this study between August 17 and December 31, 2021 [31]. The BNT162b2 and mRNA-1273 vaccines were administered 21 days and 28 days apart, respectively. Individuals with a known history of COVID-19 infection were excluded from both cohorts of patients and healthy controls. Demographic and clinical data including data for histological diagnosis, disease status, response to treatment, treatment regimen, complete blood count, and blood chemistry were obtained from medical records. The response criteria in patients with B-NHL were defined according to the Lugano response criteria for non-Hodgkin’s lymphoma [32]. The disease status in all patients was determined at the time of sample collection. We recruited healthcare workers at Aiiku Hospital who had no medical history of hematological disorders and who had received two doses of BNT162b2 vaccine as healthy controls. This study was conducted in compliance with ethical principles based on the Helsinki Declaration and was approved by the institutional review board of Aiiku Hospital. All participants provided written informed consent prior to enrollment in the study.

### Assessment of serological response

Peripheral blood and serum samples were drawn 3 months after administration of the second vaccine dose and were evaluated for anti-spike (S) SARS-CoV-2 antibodies using the Elecsys® □ Anti-SARS-CoV-2S immunoassay performed on the cobas e411 fully automated analyzer for the SARS-CoV-2 S protein receptor-binding domain [33–35]. This assay has a minimum measurement value of 0.4 U/mL, with a concentration of 0.8 U/mL or more considered to be a positive result. For individuals with an antibody titer to SARS-CoV-2 S protein of less than 0.4 U/mL, the antibody titer was calculated as 0 U/mL.

### Statistical analysis

The chi-squared test or Fisher’s exact test was used to compare categorical variables and the Mann–Whitney *U* test or an analysis of variance test was used for continuous variables. *P* values were adjusted using the Bonferroni method for multiple comparisons between each pair. We used the likelihood ratio of the receiver operator characteristics (ROC) curves and area under the curve to define the optimal cutoff for continuous variables. Univariate and multivariate logistic regression analyses were performed to evaluate potential predictors of vaccine response. Multivariate analysis with stepwise variable selection was used to assess which variables were genuinely independent. Spearman’s rank correlation coefficient was used to assess the relationship between two continuous variables. Statistical significance was determined as *P* values below 0.05, and all statistical tests were 2-sided. All statistical analyses were performed with EZR (Saitama Medical Center, Jichi Medical University, Saitama, Japan), which is a graphical user interface for R software version 2.13.0 (R Foundation for Statistical Computing, Vienna, Austria) [36].

## Results

### Characteristics of patients and healthy controls

A total of 171 patients with B-NHL as well as healthy controls (*n* = 166) were enrolled in this study. The characteristics of the patients are summarized in Tables 1 and 2. The median age of the patient cohort was 71 years (range: 31–92 years) and 87 of the patients (50.9%) were male. The healthy controls included 23 males and 143 females with a median age of 38.5 years (range: 20–72 years). A total of 140 patients and all healthy controls received BNT162b2, while 31 patients received mRNA-1273. Each patient and healthy control determined the choice of vaccine. Most patients (*n* = 158, 92.4%) had been previously exposed to anti-CD20 antibody, and the median period from the last exposure to the antibody to serology testing was 639.5 days (IQR: 163–1471.5 days). Moreover, 41 patients (24.0%), including 29 FL patients, had been previously exposed to bendamustine, and the median period from the last exposure to bendamustine to serology testing was 950 days (IQR: 719–1562 days). Within a median follow-up period of 401 days (IQR: 371.5–418.5 days) from administration of the second COVID-19 vaccine, 6 patients with B-NHL (including 2 patients who had a seronegative response, and a patient who was on therapy) developed COVID-19 infections, and most of them had mild clinical courses.

**Table 1:** Patient characteristics (*n* = 171)

Age (range)	71 (31–92)
Gender (%)	
Male	87 (50.9)
Female	84 (49.1)
Disease (%)	
DLBCL	85 (49.7)
FL	57 (33.4)
MCL	7 (4.1)
IVL	6 (3.5)
MALT	4 (2.3)
Other diseases	12 (7.0)
Disease status (%)	
CR	143 (83.6)
Non-CR	28 (16.4)
Treatment status (%)	
Off-therapy	131 (76.6)
On-therapy	31 (18.1)
Treatment-naïve	9 (5.3)
Vaccine subtype (%)	
BNT162b2	140 (81.9)
mRNA-1273	31 (18.1)
Line of therapy if on treatment, *N* (%)	
First	119 (73.5)
Second	26 (16.0)
Greater than second	17 (10.5)
Type of first-line therapy if on treatment, *N* (%)	
R-CHOP like	132 (81.5)
G-CHOP	2 (1.3)
R-B like	9 (5.5)
G-B	9 (5.5)
Rituximab	1 (0.7)
Others	9 (5.5)
Previous therapy, *N* (%)	
Anti-CD20 monoclonal antibody	158 (92.4)
Bendamustine	41 (24.0)
BTK inhibitors	2 (1.2)
CAR-T cells	1 (0.6)
HSCT	10 (5.8)

*DLBCL*, diffuse large B-cell lymphoma; *FL*, follicular lymphoma; *MCL*, mantle cell lymphoma; *IVL*, intravascular large B-cell lymphoma; *MALT*, extranodal marginal zone lymphoma of mucosa-associated lymphoid tissue; *CR*, complete remission; *BTK*, Bruton’s tyrosinekinase; *CAR-T*, chimeric antigen receptor-modified T cell; *HSCT*, hematopoietic stem cell transplantation

**Table 2:** Patient subtypes in DLBCL or FL

Cell of origin in DLBCL, *N* (%)	
GCB	38 (44.7)
Non-GCB	26 (30.6)
Not available	21 (24.7)
NCCN-IPI in DLBCL, *N* (%)	
Low	12 (14.1)
Low-intermediate	21 (24.7)
High-intermediate	28 (32.9)
High	14 (16.5)
Not available	10 (11.8)
FL grade, *N* (%)	
1	14 (24.6)
2	24 (42.1)
3A	7 (12.3)
Not available	12 (21.0)
FLIPI, *N* (%)	
Low	12 (21.1)
Intermediate	15 (26.3)
High	22 (38.6)
Not available	8 (14.0)
FLIPI2, *N* (%)	
Low	3 (5.3)
Intermediate	20 (35.1)
High	13 (22.8)
Not available	21 (36.8)

*DLBCL*, diffuse large B-cell lymphoma; *FL*, follicular lymphoma; *GCB*, germinal center B-cell-like; *NCCN-IPI*, National Comprehensive Cancer Network International Prognostic Index; *FLIPI*, Follicular Lymphoma International Prognostic Index

### Serological responses for B-NHL patients

**Fig. 1:**
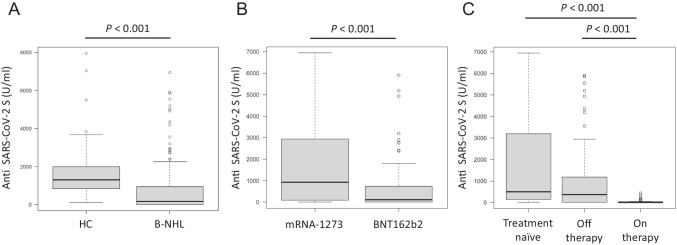
**A** Comparison of anti-SARS-CoV-2 S antibody titers between healthy controls and B-NHL patients. **B**, **C** Anti-SARS-CoV-2 S antibody titers in patients with B-NHL divided by **B** vaccine subtypes and **C** treatment status. HC, healthy controls; B-NHL, B-cell non-Hodgkin lymphoma

Patients with B-NHL showed a significantly lower seroconversion rate and a lower median antibody titer to SARS-CoV-2 S protein than those in healthy controls (70.0% vs 100%, *P* < 0.001; 166.0 U/mL, IQR: 0–943.5 vs 1301.5 U/mL, IQR: 839–1991.5, *P* < 0.001, respectively) (Fig. 1A). The seroconversion rate and median antibody titer in DLBCL patients were comparable to those in FL patients (69.4% vs 63.2%, *P* = 0.47; 124 U/mL, IQR: 0–794 vs 149 U/mL, IQR: 0–741, *P* = 0.943, respectively). The median ages of seropositive and seronegative patients with B-NHL were not significantly different (70 years, IQR: 62.5–75 vs 71 years, IQR: 66.5–75.5, *P* = 0.165). The median antibody titers did not differ statistically between male and female patients with B-NHL as well as between patients in complete remission and those in other response categories (Figs. S1A and S1B). Patients vaccinated with mRNA-1273 tended to have a higher response rate and had a significantly higher median antibody titer than those in patients vaccinated with BNT162b2 (83.9% vs 66.4%, *P* = 0.083; 928 U/mL, IQR: 95–2937 vs 114.5 U/mL, IQR: 0–731, *P* < 0.001, respectively) (Fig. 1B).

Treatment-naïve patients with B-NHL as well as those off-therapy had significantly higher response rates (100% and 74.8%, respectively) and higher median antibody titers (500 U/mL, IQR: 142–3194 and 368 U/mL, IQR: 0.9–1183.5, respectively) than those in patients on active therapy (38.7%, *P* < 0.001; 0 U/mL, IQR: 0–29, *P* < 0.001, respectively) (Fig. 1C). The serologic response rates and median antibody titers did not differ significantly between patients who received one line of therapy and patients who received two or more lines of therapy as well as between patients with B-NHL who underwent HSCT and those who did not undergo HSCT (Figs. S1C and S1D). Among the patients with DLBCL or FL, the median antibody titers in the different types and risks are shown in Fig. S2. None of them had a statistically significant impact on median antibody titers except for FLIPI2 between low and intermediate (1768 U/mL, IQR: 1254.5–4355 vs 2.64 U/mL, IQR: 0–144, *P* = 0.009).

### Factors affecting serological responses in B-NHL patients

**Fig. 2:**
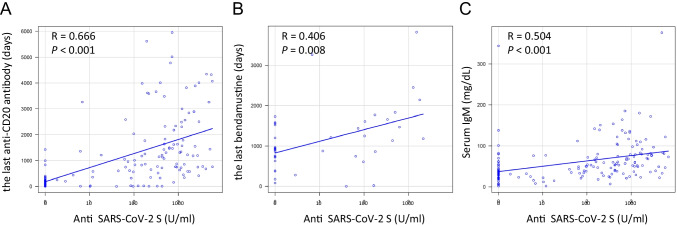
Correlations of anti-SARS-CoV-2 S antibody titers with **A** the period from the last anti-CD20 antibody treatment to vaccination, **B** the period from the last bendamustine treatment to vaccination and **C** serum IgM level

In univariate analysis, the variables that were significantly associated with a lack of serological response included a period of less than 11 months from the last anti-CD20 antibody treatment to vaccination (odds ratio (OR) = 74.8, 95% confidence interval (CI): 21.1–266), previous treatment with bendamustine (OR = 2.915, 95% CI: 1.404–6.061), hemoglobin level < 12.0 g/dL (OR = 3.00, 95% CI: 1.52–5.92), serum IgA level < 166 mg/dL (OR = 5.14, 95% CI: 2.41–11.0), serum IgG level < 932 mg/dL (OR = 2.89, 95% CI: 1.47–5.71), serum IgM level < 46 mg/dL (OR = 7.65, 95% CI: 3.55–16.5), absolute lymphocyte count < 1232 cells/mL (OR = 3.91, 95% CI: 1.97–7.76), absolute neutrophil count < 2585 cells/mL (OR = 2.17, 95% CI: 1.11–4.25), and platelet count < 187,000 cells/mL (OR = 2.52, 95% CI: 1.29–4.92), based on ROC analysis (Table 3). In multivariate analysis, the independent variables associated with response included a period of less than 11 months from the last anti-CD20 antibody treatment to vaccination (OR = 85.6, 95% CI: 22.3–329.0) and previous treatment with bendamustine (OR = 3.89, 95% CI: 1.14–13.2) (Table 3). Moreover, antibody titers showed correlations with the period from the last anti-CD20 antibody treatment to vaccination (*R* = 0.666, *P* < 0.001), the period from the last bendamustine treatment to vaccination (*R* = 0.406, *P* = 0.008), and serum IgM level (*R* = 0.504, *P* < 0.001) (Fig. 2).

**Table 3:** Results of univariate and multivariate analyses of seroconversion among patients with B-NHL

Seroconversion		Univariate		Multivariate	
Factor		Hazard ratio (95% CI)	*P* value	Hazard ratio (95% CI)	*P* value
Age	< 66 vs. ≥ 66 years	0.492 (0.229–1.060)	0.069		
Gender	Male vs. female	0.763 (0.396–1.464)	0.415		
Disease	DLBCL vs. the others	1.000 (0.521–1.920)	1.000		
Disease status	CR vs. non-CR	0.909 (0.380–2.165)	0.827		
Line of therapy	1 vs. ≥ 2	0.576 (0.279–1.190)	0.135		
Vaccine subtype	BNT162b2 vs. mRNA-1273	2.624 (0.952–7.299)	0.063		
Period from the last anti-CD20 antibody treatment to vaccination	< 11 vs. ≥ 11 months	74.80 (21.10–266.0)	< 0.001	85.60 (22.30–329.0)	< 0.001
Previous therapy with bendamustine	Yes vs. no	2.915 (1.404–6.061)	0.004	3.890 (1.140–13.20)	0.029
Previous therapy of HSCT	Yes vs. no	0.546 (0.111–2.659)	0.453		
Serum IgA	< 166 vs. ≥ 166 mg/dL	5.140 (2.410–11.00)	< 0.001		
Serum IgG	< 932 vs. ≥ 932 mg/dL	2.890 (1.470–5.710)	0.003		
Serum IgM	< 46 vs. ≥ 46 mg/dL	7.650 (3.550–16.50)	< 0.001		
Absolute neutrophil count	< 2585 vs. ≥ 2585 cells/mL	2.170 (1.110–4.250)	0.025		
Absolute lymphocyte count	< 1232 vs. ≥ 1232 cells/mL	3.910 (1.970–7.760)	< 0.001		
Absolute monocyte count	< 372 vs. ≥ 372 cells/mL	0.606 (0.310–1.190)	0.144		
Hemoglobin	< 12.0 vs. ≥ 12.0 g/dL	3.000 (1.520–5.920)	0.002		
Platelet count	< 187,000 vs. ≥ 187,000 cells/mL	2.520 (1.290–4.920)	0.006		

*B-NHL*, B-cell non-Hodgkin lymphoma; *DLBCL*, diffuse large B-cell lymphoma; *CR*, complete remission; *HSCT*, hematopoietic stem cell transplantation

### Serological responses to anti-CD20 antibody administration in DLBCL and FL patients

**Fig. 3:**
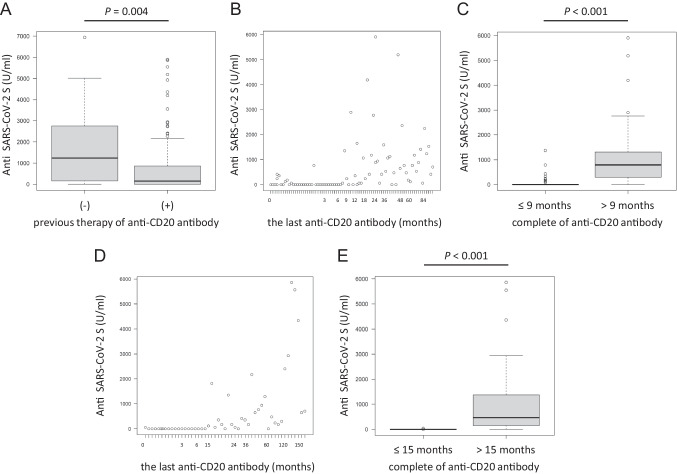
**A** Anti-SARS-CoV-2 S antibody titers in B-NHL patients divided by previous therapy with anti-CD20 antibody. **B** Scatter plot of the period from the last anti-CD20 antibody treatment to vaccination and anti-SARS-CoV-2 S antibody titers in patients with DLBCL. **C** Comparison of anti-SARS-CoV-2 S antibody titers between DLBCL patients who completed anti-CD20 antibody treatment within 9 months before vaccination and those who completed the treatment more than 9 months before vaccination. **D** Scatter plot of the period from the last anti-CD20 antibody treatment to vaccination and anti-SARS-CoV-2 S antibody titers in patients with FL. **E** Comparison of anti-SARS-CoV-2 S antibody titers between FL patients who received the last anti-CD20 antibody treatment within 15 months prior to vaccination and those who received the treatment more than 15 months prior to vaccination

Patients who had been previously exposed to anti-CD20 antibody showed a significantly lower seroconversion rate and a lower median antibody titer than those in patients who had never received anti-CD20 therapy (67.1% vs 100%, *P* = 0.01; 149.5 U/mL, IQR: 0–846 vs 1240 U/mL, IQR: 161–2756, *P* = 0.004, respectively) (Fig. 3A). Based on ROC analysis, the serologic response rates and median antibody titers were significantly different between DLBCL patients who completed anti-CD20 antibody treatment within 9 months before vaccination (*n* = 41) and those who completed the treatment more than 9 months before vaccination (*n* = 43) (39.0% vs 97.7%, *P* < 0.001; 0 U/mL, IQR: 0–11.5 vs 790 U/mL, IQR: 296.5–1315, *P* < 0.001, respectively) (Fig. 3B and C). Similarly, the serologic response rates and median antibody titers were significantly different between FL patients who received the last anti-CD20 antibody treatment within 15 months prior to vaccination (*n* = 20) and those who received the last treatment more than 15 months prior to vaccination (*n* = 30) (5.0% vs 93.3%, *P* < 0.001; 0 U/mL, IQR: 0–0 vs 462 U/mL, IQR: 157.5–1348, *P* < 0.001, respectively) (Fig. 3D and E).

### Serological responses to bendamustine administration in FL patients

**Fig. 4:**
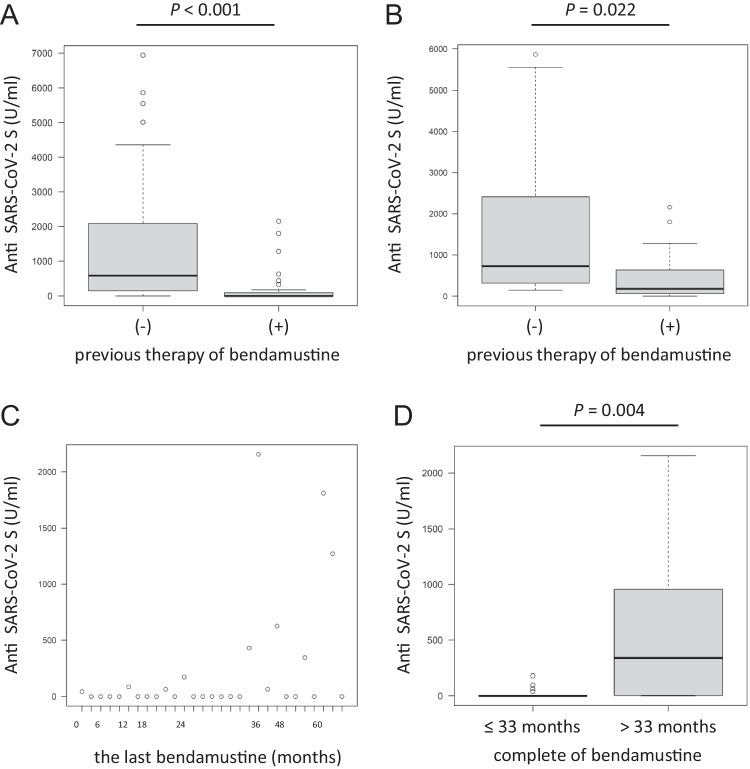
**A**, **B** Anti-SARS-CoV-2 S antibody titers in FL patients divided by previous therapy with bendamustine in **A** the entire cohort and **B** the cohort of patients who completed anti-CD20 antibody treatment more than 15 months before vaccination. **C** Scatter plot of the period from the last bendamustine treatment to vaccination and anti-SARS-CoV-2 S antibody titers in patients with FL. **D** Comparison of anti-SARS-CoV-2 S antibody titers between FL patients who completed bendamustine treatment within 33 months before vaccination and those who completed the treatment more than 33 months before vaccination

Furthermore, FL patients who had previously received bendamustine had a significantly lower seroconversion rate and a lower median antibody titer than those in FL patients who had never received bendamustine (41.4% vs 85.7%, *P* < 0.001; 0 U/mL, IQR: 0–96.5 vs 583 U/mL, IQR: 149.5–1928.5, *P* < 0.001, respectively) (Fig. 4A). Among the FL patients who received the last anti-CD20 antibody treatment more than 15 months prior to vaccination (*n* = 30), patients who had previously received bendamustine also had a significantly lower median antibody titer than those who had never received bendamustine, although the serologic response rates did not differ statistically (84.6% vs 100%, *P* = 0.179; 180 U/mL, IQR: 64.8–630 vs 724 U/mL, IQR: 316–2410, *P* = 0.022, respectively) (Fig. 4B). Based on ROC analysis, the serologic response rates and median antibody titers were significantly different between FL patients who completed bendamustine treatment within 33 months before vaccination (*n* = 18) and those who completed the treatment more than 33 months before vaccination (*n* = 11) (22.2% vs 72.7%, *P* = 0.017; 0 U/mL, IQR: 0–0.5 vs 339 U/mL, IQR: 3.5–956, *P* = 0.004, respectively) (Fig. 4C and D).

## Discussion

In the current study, the serologic responses to two doses of COVID-19 vaccine in B-NHL patients were evaluated and compared with those in age-compatible vaccinated healthy controls. Effective vaccination against SARS-CoV-2 would contribute to the protection of patients against COVID-19. However, consistent with previous reports, patients with B-NHL who had previously been exposed to treatment had a poor humoral response to COVID-19 vaccination due to inherent immune deficiency and therapy-related immune suppression [23–30]. Median antibody titers had a statistically significant impact between FLIPI2 low and FLIPI2 intermediate. The percentage of treatment-naïve patients was higher in FLIPI2 low patients than FLIPI2 intermediate patients (66.7% vs 10.0%, *P* = 0.07). In contrast, the percentage of treatment-naïve patients was not different significantly among the types and risks shown in Fig. S2, except for between FLIPI2 low and FLIPI2 intermediate (data not shown).

In our cohort, the main predictive factors influencing antibody levels following vaccination were the period from the last anti-CD20 antibody treatment to vaccination, the period from the last bendamustine treatment to vaccination and serum IgM level. Previous studies showed that CD19-positive lymphocyte count in peripheral blood and serum IgM titer correlated significantly with the acquired antibody titer in patients treated with anti-CD20 antibodies [23, 29, 30]. IgM titer increases within the first 2 weeks in response to an antigen such as a virus, followed by degradation over a period of several weeks, and low serum IgM titer is a manifestation of B-cell depletion [37].

From the viewpoint of therapy-related factors, patients with DLBCL and FL who received anti-CD20 antibodies within 9 months and 15 months before vaccination failed to produce anti-spike antibodies against COVID-19. The recovery of peripheral blood B-cell depletion after anti-CD20 therapy starts at 6 months after treatment with a return to normal counts at 12 months [38, 39]. In our study, 6 patients of FL received anti-CD20 antibody maintenance therapy among those on therapy. The median number of anti-CD20 antibody treatment was higher in FL patients than those in DLBCL patients (15 vs 6, *P* < 0.001); however, the number of anti-CD20 antibody treatment did not correlate significantly with the acquired antibody titer, and did not differ significantly between seropositive and seronegative patients among those on therapy (*R* =  − 0.309, *P* = 0.124; 7 vs 8, *P* = 0.112). A previous study showed that the median period to achieve positive serology was prolonged in patients with indolent B-NHL compared with that in patients with aggressive B-NHL, and this difference probably reflects the deeper immune suppression imposed by longer exposure to anti-CD20 antibodies and incurable status in patients with indolent B-NHL [26]. In our study, however, all of treatment-naïve patients with indolent B-NHL (*n* = 8) achieved seroconversion, and the median period from the last exposure to anti-CD20 antibodies did not differ significantly between patients with DLBCL and patients with FL (424 days, IQR: 147.5–1345 vs 831 days, IQR: 180.5–1753, *P* = 0.102).

Our results also showed a poor response to COVID-19 vaccination in the setting of bendamustine, particularly in FL patients. To the best of our knowledge, our study is the first study showing the effects of bendamustine on humoral response to COVID-19 vaccine for B-NHL patients in a prospective cohort. We speculated that bendamustine administration was the reason for the difference in the periods from the last anti-CD20 antibody treatment to vaccination between DLBCL and FL patients. Previous studies showed that CD4-positive lymphocyte count in peripheral blood correlated significantly with the acquired antibody titer in patients treated with anti-CD20 antibodies, suggesting that CD4-positive T cells established by COVID-19 vaccination are necessary for the generation of high-affinity antibodies and development of memory B-cells and may contribute to long-term memory immunity against SARS-CoV-2 [30, 40–44]. Since it has been reported that bendamustine reduced CD4-positive T cells, we speculated that bendamustine might impede the serologic response [45]. In fact, there have been several studies on B-NHL patients with COVID-19 severity and mortality who were previously exposed to both anti-CD20 antibody and bendamustine [46–50]. Our results also suggested that these immunocompromised patients have a high risk of COVID-19 infection.

Our study has several limitations. There were differences between lymphoma patients and healthy controls in age, sex, and mRNA vaccine. Although we studied the protective impact of vaccination and its ability to prevent SARS-CoV-2 infection or clinically significant COVID-19 in B-NHL patients, further investigations with a larger cohort are required. We instructed the patients who had a high risk of COVID-19 infection to take precautions such as social distancing. Therefore, we speculated that only 6 patients developed COVID-19 infections.

## Conclusion

In summary, our study demonstrated that B-NHL patients who were recently treated with anti-CD20 antibodies or bendamustine had a diminished humoral response to COVID-19 vaccination and that serum IgM titer is a biomarker to predict the antibody response. We concluded that depletion of B cells or T cells was impaired humoral response to COVID-19 vaccination.

## Supplementary Information

Below is the link to the electronic supplementary material.

Supplementary file1 (PDF 456 KB)

## Data Availability

The data that support the findings of this study are available from the corresponding author upon reasonable request.
