# Supplementary figures and images for: Anti-CD20 antibodies and bendamustine attenuate humoral immunity to COVID-19 vaccination in patients with B-cell non-Hodgkin lymphoma
Source: Ann Hematol. 2023 Apr 12;102(6):1421–31. doi: 10.1007/s00277-023-05204-7 (PMC10089694; doi:10.1007/s00277-023-05204-7)

Figure S1

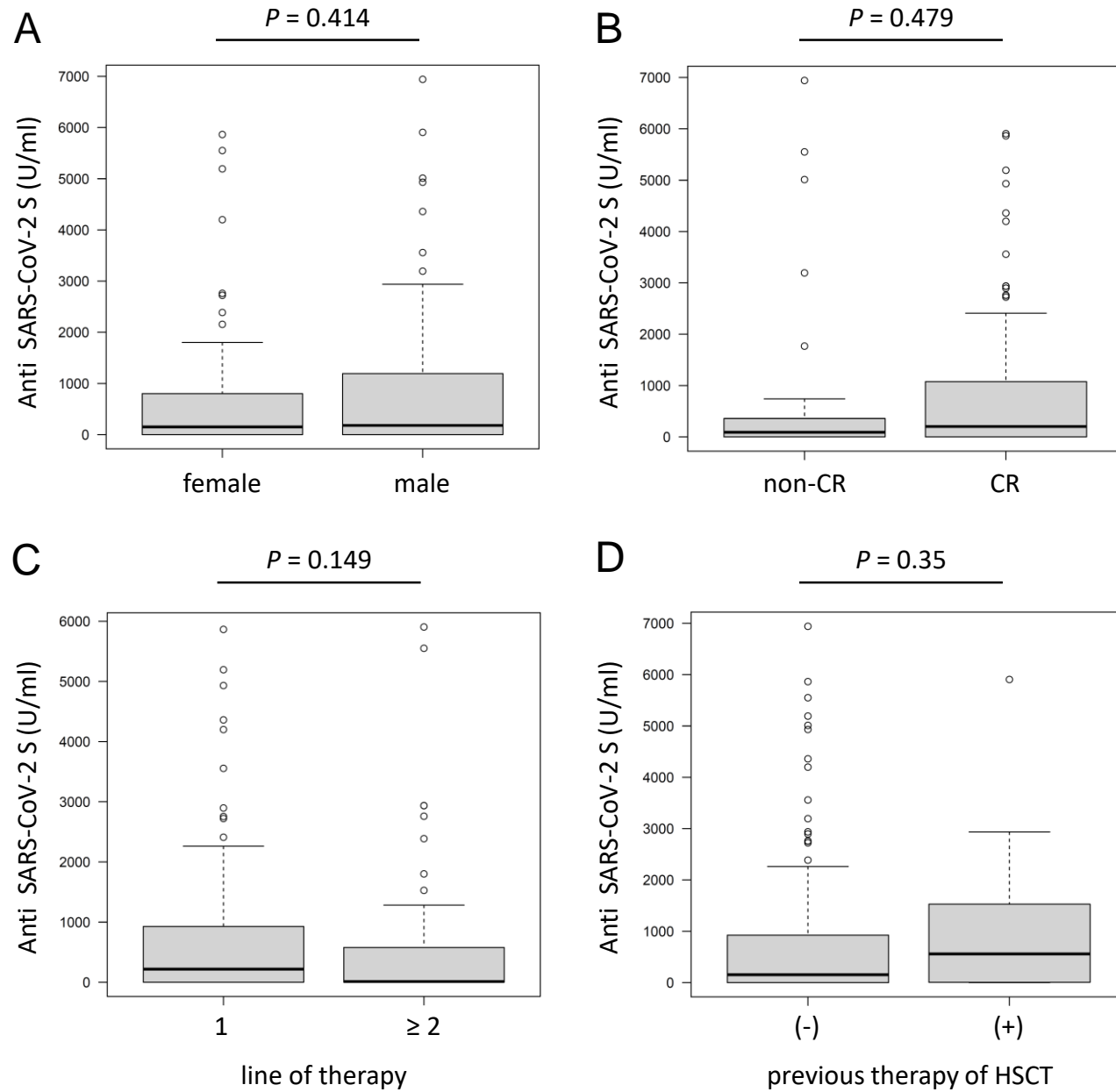

Figure S2

A

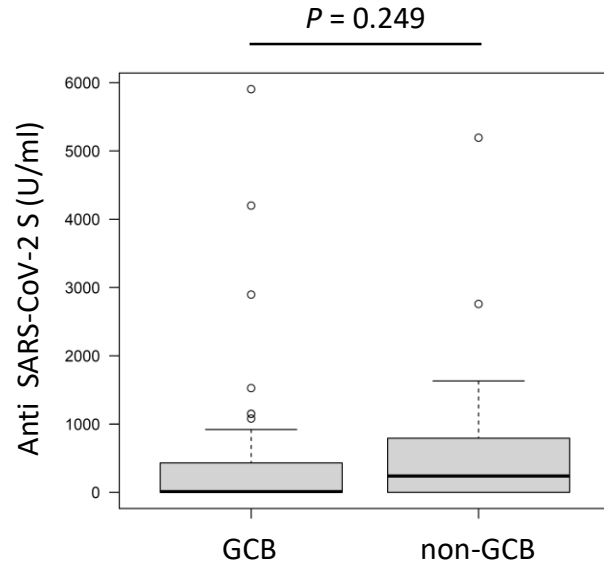

B

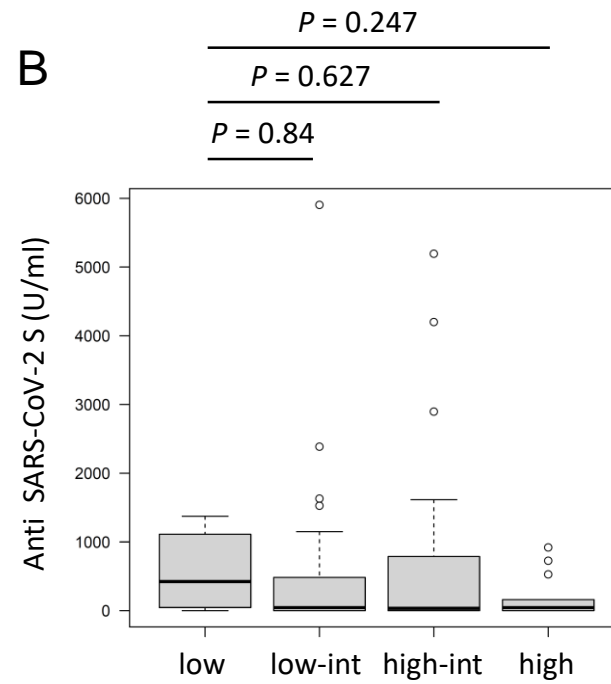

C

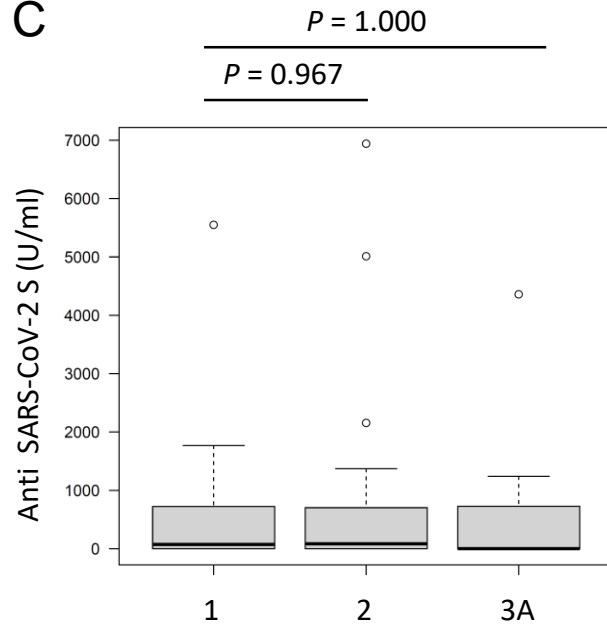

D

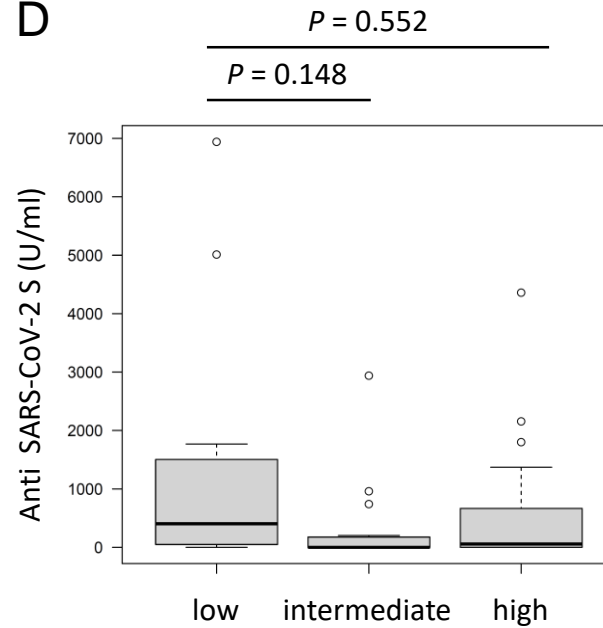

E

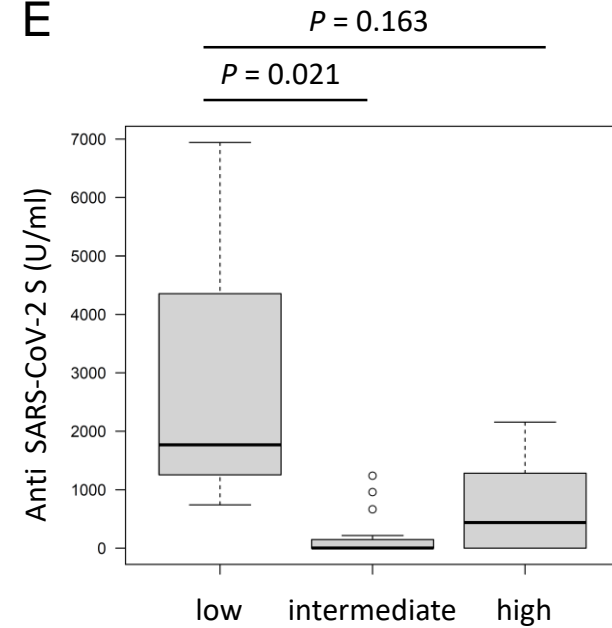

Supplement: Supplementary file 1 — Supplementary file1 (PDF 456 KB) [file 277_2023_5204_MOESM1_ESM.pdf]
